# Recent advances in managing septal defects: ventricular septal defects and atrioventricular septal defects

**DOI:** 10.12688/f1000research.14102.1

**Published:** 2018-04-26

**Authors:** P Syamasundar Rao, Andrea D Harris

**Affiliations:** 1 University of Texas-Houston McGovern Medical School, Children Memorial Hermann Hospital, Houston, USA; 2 Pediatrix Cardiology Associates of New Mexico, Albuquerque, USA

**Keywords:** Ventricular septal defect, atrioventricular septal defect, surgery, percutaneous treatment, hybrid procedure

## Abstract

This review discusses the management of ventricular septal defects (VSDs) and atrioventricular septal defects (AVSDs). There are several types of VSDs: perimembranous, supracristal, atrioventricular septal, and muscular. The indications for closure are moderate to large VSDs with enlarged left atrium and left ventricle or elevated pulmonary artery pressure (or both) and a pulmonary-to-systemic flow ratio greater than 2:1. Surgical closure is recommended for large perimembranous VSDs, supracristal VSDs, and VSDs with aortic valve prolapse. Large muscular VSDs may be closed by percutaneous techniques. A large number of devices have been used in the past for VSD occlusion, but currently Amplatzer Muscular VSD Occluder is the only device approved by the US Food and Drug Administration for clinical use. A hybrid approach may be used for large muscular VSDs in small babies. Timely intervention to prevent pulmonary vascular obstructive disease (PVOD) is germane in the management of these babies. There are several types of AVSDs: partial, transitional, intermediate, and complete. Complete AVSDs are also classified as balanced and unbalanced. All intermediate and complete balanced AVSDs require surgical correction, and early repair is needed to prevent the onset of PVOD. Surgical correction with closure of atrial septal defect and VSD, along with repair and reconstruction of atrioventricular valves, is recommended. Palliative pulmonary artery banding may be considered in babies weighing less than 5 kg and those with significant co-morbidities. The management of unbalanced AVSDs is more complex, and staged single-ventricle palliation is the common management strategy. However, recent data suggest that achieving two-ventricle repair may be a better option in patients with suitable anatomy, particularly in patients in whom outcomes of single-ventricle palliation are less than optimal. The majority of treatment modes in the management of VSDs and AVSDs are safe and effective and prevent the development of PVOD and cardiac dysfunction.

## Introduction

Septal defects are the most common types of congenital heart defects (CHDs) with the exception of bicuspid aortic valve. Advances in the management of atrial septal defects (ASDs) have been addressed in an earlier review ^1^. In the current article, ventricular septal defects (VSDs) and atrioventricular septal defects (AVSDs) will be discussed. Whereas transcatheter (percutaneous) approaches are the mainstay in the management of secundum ASDs, the VSDs and AVSDs are at present managed largely by surgical methodology since a limited number of lesions are amenable for transcatheter and hybrid approaches. In this review, we will present a classification of the VSDs and AVSDs, indications for repair, and a discussion of surgical, transcatheter, and hybrid methodologies with a particular focus on advances in the management of these defects.

## Ventricular septal defects

Isolated VSDs are the most common CHDs (provided that subjects with bicuspid aortic valve are excluded) and constitute 20 to 25% of all CHDs. They are most commonly classified on the basis of their location in the ventricular septum and are divided into perimembranous (situated in the membranous ventricular septum in the subaortic region), supracristal (found in the conal septum in the subpulmonary region), atrioventricular (AV) septal (defect located in the posterior septum), and muscular (located in the muscular and apical areas of the ventricular septum) ^2– 5^. The membranous defects are most common among the VSDs (80% prevalence), and supracristal (5 to 7%), AV septal (8%), and muscular (5 to 20%) defects are much less common. These defects may be large, medium, or small in size. Most of the defects are single; however, multiple defects may be present in the muscular septum, described as the “Swiss cheese” type of VSDs.

Left-to-right shunt across the VSD produces dilatation of the left atrium and left ventricle (LV). Owing to high pulmonary vascular resistance (PVR), this shunt may not manifest in the neonate and during the first weeks of life. As the pulmonary arterioles involute, PVR falls with resultant increase in left-to-right shunt. The right ventricle (RV) and main and branch pulmonary arteries may also be dilated in moderate to large defects. Whereas pulmonary vascular obstructive disease (PVOD) does not manifest until adulthood in patients with ASD, patients with VSD are likely to develop PVOD as early as 18 months to 2 years of age if a large VSD is left unrepaired.

Discussion of VSDs seen in association with tetralogy of Fallot, pulmonary atresia/stenosis, transposition of the great arteries, tricuspid and mitral atresia, and double-outlet RV and heterotaxy (asplenia and polysplenia) syndromes will not be included in this article. Similarly, post-traumatic and post-myocardial infarction VSDs will not be addressed in this review.

### Indications for ventricular septal defect closure

The indications for intervention depend, to a large degree, on the size and type of the VSD ^2– 6^. Closure of the VSD is not necessary in patients with a small VSD. Assurance of the parents and perhaps subacute bacterial endocarditis prophylaxis and occasional clinical follow-up are suggested. However, if the VSD has become smaller because of its closure by prolapsed aortic valve cusp into the defect with resultant aortic insufficiency, surgical closure of the defect with resuspension of the aortic valve leaflets is recommended. The development of aortic insufficiency is seen in both membranous and supracristal VSDs.

In moderate-sized VSDs, congestive heart failure (CHF), if present, should be treated. In the presence of failure to thrive, markedly enlarged left atrium and LV or elevated pulmonary artery pressures (or both), closure of the defect is generally recommended. An additional criterion is a pulmonary-to-systemic flow ratio (Qp:Qs) greater than 2:1.

In large VSDs with systolic pressures in the RV and pulmonary artery close to left ventricular and aortic systolic pressures, closure should be undertaken. This should be done prior to 6 to 12 months of age (certainly no later than 18 months of age) irrespective of control of heart failure and adequacy of weight gain. The reason for this recommendation is to prevent irreversible PVOD. In babies with Down syndrome, such closure should be undertaken prior to six months of age since these patients tend to develop PVOD sooner than non-Down babies. In present-day practice, primary surgical correction is preferred in contradistinction to initial pulmonary artery banding followed by surgical closure of the VSD, a common practice in an earlier era. However, such a staged approach may be considered for the Swiss-cheese variety of muscular VSDs. Some clinicians may also use pulmonary artery banding in low-weight infants who may have an increased risk of heart block with repair, although the current trend is toward early complete repair.

In cases with elevated PVR, most authorities would suggest intervention if the calculated PVR index is less than 6 Wood units or the pulmonary-to-systemic vascular resistance ratio (Rp:Rs) is less than 0.35 (or both) with a Qp:Qs greater than 1.5 ^6– 8^. In patients with higher PVR values, pulmonary vascular reactivity testing with oxygen and nitric oxide (NO) should be performed ^6, 8, 9^. If the PVR index drops below 6 to 8 units with oxygen or NO, then these patients become candidates for VSD closure.

Patients with large VSD and severely elevated PVR (that is, irreversible PVOD) are not suitable for VSD closure. These patients eventually may become candidates for lung transplantation.

### Management

The treatment of the VSD, as mentioned above, is largely dependent on its size and the clinical status of the patient. Several methods of available treatment options will be reviewed.

***Medical management.*** Infants with moderate to large VSDs with signs of CHF should receive aggressive treatment with anti-congestive measures to include digoxin, diuretics (furosemide and aldactone), and afterload reducing agents (angiotensin-converting enzyme inhibitors: captopril/enalopril and others). Although the above order of drug administration has been used for a long time, recent doubts about the usefulness of digoxin have changed the order of administration to diuretics, afterload reducing agents, and digoxin (in that order). We continue to believe that digoxin is useful in the management of CHF in infants and children. When chronic administration of furosemide is required, aldactone may be added for its potassium-sparing effect. Optimization of nutrition, maintenance of adequate hemoglobin level, and appropriately addressing the associated respiratory symptoms should be a part of overall management of these babies.

Clinical improvement may occur with adequate medical therapy; such improvement may be related to spontaneous closure or diminution in the size of the VSD, development of right ventricular outflow tract obstruction (Gasul’s transformation), or increased PVR. Careful clinical and echocardiographic follow-up evaluation and, when necessary, cardiac catheterization should be undertaken to ensure that the improvement is not secondary to elevation of PVR. As mentioned in the preceding section, VSD closure should be undertaken prior to 18 months of age to prevent the development of irreversible PVOD.

As also mentioned in the previous section, large VSDs with severe increase of PVR (irreversible PVOD) should not have their VSDs closed. Recently described pulmonary vasodilators (prostacyclins, sildenafil, bosentan, and others), which may improve symptoms, may be used. If severe polycythemia is present, erythropheresis to treat symptoms of polycythemia should be performed. Transcatheter creation of atrial communication may relieve symptoms of PVOD. Discussion of the pulmonary vasodilators and management of PVOD is beyond the scope of this article. Ultimately, patients with PVOD may need lung transplantation.

Although it is extremely important that every effort be made to prevent PVOD, it should be recognized that 40% of the VSDs close spontaneously and an additional 25 to 30% of defects become small enough not to require intervention. Defects in the muscular septum tend to close more often than the defects in the perimembranous region. Small defects are likely to close more often than large VSDs (60% versus 20%). It is well documented that even large defects producing CHF or requiring pulmonary artery banding during infancy close spontaneously. Whereas most of the defects close by two years of age, the process of spontaneous closure continues through childhood, adolescence, and adulthood. These considerations should be kept in mind when decisions to recommend surgical or transcatheter closure of VSDs are being made.

***Surgical repair.*** Following the description of cardiopulmonary bypass techniques by Gibbon, Lillehei, and Kirklin in the 1950s to successfully close ASDs ^1^, surgical techniques for repair of other CHDs, including VSDs, were soon developed. Median sternotomy incision is performed under general anesthesia and the aorta and vena cavae are cannulated to institute cardiopulmonary bypass. The majority of perimembranous VSDs are closed with a Dacron patch via right atriatomy with or without detachment of the tricuspid valve leaflets. Supracristal defects are addressed via the pulmonary valve. VSDs with associated aortic insufficiency, though small, should be closed to prevent progression of aortic insufficiency ^10^. Moderate to severe aortic valve prolapse may require re-suspension of the aortic valve or other valvuloplasty techniques or both ^11, 12^.

Small muscular VSDs are likely to close spontaneously and do not require surgery. In infants, large muscular VSDs, particularly of the “Swiss cheese” variety, are difficult to close from the right ventricular side. Pulmonary artery banding initially to control CHF and reduce the pulmonary artery pressures is performed in young (up to three months of age) babies. Closure of the VSD via an apical left ventriculotomy may be performed later during childhood ^13, 14^. At that time, the pulmonary artery band is removed and the pulmonary artery is repaired, if necessary, to ensure that there is no residual stenosis at the prior band site. The senior author (PSR) observed several patients in whom the muscular VSD closed spontaneously following pulmonary artery band placement, and repeat thoracotomy to remove the pulmonary band was required. Absorbable pulmonary artery band may be a good option in such situations. The polydioxanone band (absorbable) reduces pulmonary blood flow and pressure initially and helps decrease symptoms of CHF. When the VSD closes spontaneously, the pulmonary artery band is also resorbed and will not require additional surgery to remove it. The principles are similar to those advocated for patients with tricuspid atresia with a large VSD ^15, 16^. Unfortunately, however, most surgeons are reluctant to use an absorbable pulmonary artery band in this situation.

**Results**

Surgical closure of VSDs is safe; the mortality rate is less than 3%. The long-term outlook following surgery in both the early era ^17^ and more recent times ^18^ is generally good with rare residual shunts, frequent right bundle branch block, occasional pulmonary hypertension, infrequent heart block or sinus node dysfunction, and modest progression of aortic insufficiency. Following VSD closure, the left ventricular volume and mass return to normal with preservation of normal left ventricular function ^19^.

***Percutaneous closure.*** Percutaneous closure of VSDs in animal models was first reported by Rashkind in the early 1970s ^20^. He used hooked, single-disc, and double-disc Rashkind devices. Afterwards, other cardiologists followed his lead and used Rashkind’s double-disc patent ductus arteriosus (PDA) umbrella, Rashkind’s ASD double-umbrella, and clamshell devices for transcatheter occlusion of VSDs ^21– 26^. Subsequently, other devices were used to percutaneously occlude the VSDs: buttoned device (Custom Medical Devices, Amarillo, TX, USA) ^27, 28^, CardioSEAL and STARFlex devices (Nitinol Medical Technologies, Inc., Boston, MA, USA) ^29^, Nit-Occlud device (pfm - Produkte für die Medizin AG, Köln, Germany) ^30– 33^, Amplatzer Muscular VSD Occluder device (St. Jude Medical, Inc., St. Paul, MN, USA) ^34– 39^, detachable steel coils (Cook, Bloomington, IN, USA) ^40^, Gianturco coils (Cook) ^41^, flipper, 0.052ʺ Gianturco, or 0.035ʺ platinum coils (Cook) ^42^, wireless devices such as detachable balloon and transcatheter patch (Custom Medical Devices) ^43^, Amplatzer Duct Occluder (St. Jude Medical, Inc.) ^44^, Shanghai symmetrical perimembranous VSD occluder (Shape Memory Alloy Ltd., Shanghai, China) ^45^, Amplatzer Duct Occluder II (St. Jude Medical, Inc.) ^46– 56^, Cera devices (Lifetech Scientific Co. Ltd., Shenzhen, China) ^57^, and perhaps other devices (not known to the authors).

The majority of the above mentioned (with exception of coils and Amplatzer Duct Occluder) are double-disc devices and require septal rims to hold the device in place. Therefore, they can be used only in occluding muscular VSDs and perimembranous defects with a good-sized aortic rim. Owing to lack of aortic rim and proximity of the aortic valve to the defect, it may not be feasible to close the more common perimembranous VSDs. In an attempt to address this challenge, the device was redesigned ^58^ so that the aortic end of the left ventricular disc is made to be shorter (0.5 mm) while the other end is designed to be longer (5.5 mm). The lower pole of the left ventricular disc was impregnated with a platinum marker in order to aid appropriate device positioning during deployment of the device. This redesigned device, now called Amplatzer Membranous VSD Occluder (St. Jude Medical, Inc.), was used in patients with small- to medium-sized VSDs ^59– 68^, including in US Food and Drug Administration (FDA)-approved US clinical trials ^67^. The results were generally considered acceptable. In addition to the usual complications seen with complex procedures, complete heart block ^60– 65, 67– 76^ was detected both immediately after and during follow-up after implantation of Amplatzer Membranous VSD occluders, raising concerns ^77, 78^ regarding the advisability of using this device.

The connecting waist of most double-disc devices is located within the VSD, is small, and is not likely to stretch the defect. The mechanism of defect closure is by stop-flow by the discs on both sides of the VSD. However, the Amplatzer Membranous VSD Occluder indeed “stents” the defect and, with time, stretches the defect since the device size is generally larger than the size of the VSD. Since the conduction system is situated along the rims of the VSD, the device possibly exerts pressure on the conduction system ^78^. This is likely to be the mechanism for reported development of heart block ^60– 65, 67– 76^ and conduction abnormalities ^63– 65, 71, 72^. The described incidence of complete heart block varied between 1 and 22% and many of them required pacemaker therapy ^60– 65, 67– 76^. This is in contradistinction to 1% incidence of heart block following surgical closure ^79^. Therefore, it is difficult to justify the use of membranous VSD Occluder to transcatheter-occlude the VSDs. It has been suggested that the device be redesigned to make its edges supple or soft such that less or no pressure is exerted on the conduction system ^78^. However, to the best of authors’ knowledge, no such modifications seem to have been undertaken by the device manufacturer.

Most of the devices described above had experimental device closures in animal models. Clinical trials in human subjects followed with local institutional review board, CR Mark (in Europe), or FDA (in the USA) approval. Feasibility, safety, and effectiveness of occluding the VSDs have been shown for the majority of the devices. However, at this time, only the Amplatzer Muscular VSD Occluder has received approval for clinical use by the FDA. Although CardioSEAL Septal Occlusion System with QwikLoad and the STARFlex Septal Occlusion System have received FDA approval in the past, they are not widely used at present. Free and detachable Gianturco coils ^41, 42^, Nit-Occlud device ^30– 33, 75^, Amplatzer Duct Occluder ^44^, and Amplatzer Duct Occluder II ^46– 56^ are also being used on an off-label basis. Discussion of devices used on an off-label basis will not be included in this review; the interested reader may review the respective publications ^30– 33, 41, 42, 44, 46– 56, 75^.

***Amplatzer muscular ventricular septal defect occluder.***

**Description of the device**

The device is constructed with 0.004ʺ to 0.005ʺ Nitinol (nickel–titanium compound) wire with shape memory and consists of two equal-sized discs connected with a 7-mm-long waist. The discs on either side of the waist are 4 mm longer than the waist. Dacron polyester patches are sewn into both discs. The device size is determined by the diameter of the connecting waist. Available device sizes are 4 to 18 mm. The device is easily retrieved and redeployed.

**Method of implantation**

Following recording of the usual hemodynamic data, a biplane left ventricular cine-angiogram is performed in a sitting-up (60° long-axis oblique and 30° cranial) and lateral views to define the size and location of the VSD. Balloon sizing of the VSD, performed at the beginning, is no longer routinely performed; instead, transesophageal echocardiographic (TEE) and angiographic diameters are used to measure the VSD size. Additional angiography in other views is obtained if the required data on the VSD size and location could not be clearly defined. Initially, a right coronary artery, multipurpose, or balloon wedge catheter is advanced from the LV into the RV via the VSD with the aid of a soft-tipped guide wire, and an exchange-length guide wire (Noodle wire, St. Jude Medical, Inc.) is passed and its tip is positioned either in the superior vena cava or in the pulmonary artery. The tip of the wire is snared from the femoral venous route and the wire exteriorized, establishing an arterio-venous guide-wire loop. Now, the selected delivery sheath is advanced from the femoral vein and positioned into the left ventricular apical region and the guide wire and catheters are removed. A right internal jugular venous approach may be required in some cases, especially when kinking of the sheath introduced via the femoral vein occurs. Alternatively, the device may be delivered from the arterial route. A pigtail catheter is placed retrogradely into the LV. An Amplatzer Muscular VSD Occluder that is 1 to 2 mm larger than the diameter of the VSD is selected for deployment. The device is screwed onto a delivery wire and loaded into the delivery sheath while taking precautions to eliminate air bubbles in the system. The device is advanced within the delivery sheath, and the left ventricular disc is delivered into the LV under fluoroscopic and TEE guidance. After making sure that the device does not impinge on the mitral valve apparatus, the left ventricular disc is pulled back against the ventricular septum under fluoroscopic and TEE guidance. If necessary, left ventricular angiography is performed to verify the position of the device. The waist of the device is delivered into the defect. Then the right ventricular disc is deployed on the right side of the ventricular septum by slow withdrawal of the sheath, again while monitoring by fluoroscopy and TEE. Left ventricular angiography and TEE are repeated to ensure good position of the device which is still connected to the delivery wire. At this point, the device is still retrievable back into the delivery sheath and redeployed as seen fit. Once the device position is satisfactory, the device is disconnected from the delivery wire. A final left ventricular angiogram and TEE are performed prior to removal of the catheters and sheaths. If additional muscular VSDs are present, they may also be occluded by placement of additional Amplatzer Muscular VSD Occluders as previously described elsewhere ^37, 80^. Administration of heparin (100 units/kg and additional doses to maintain activated clotting times above 200 seconds), antibiotics (usually Ancef, three doses eight hours apart) and anti-platelet therapy (aspirin or clopidogrel or both), in accordance with institutional protocol for intracardiac device implantations, is undertaken.

**Results**

Immediate results of implantation of Amplatzer Muscular VSD Occluders in 1 to 119 patients have been reported ^34– 39, 74, 80– 83^. Most reports involving single-institution studies stated that some degree of contrast foaming through the device was seen immediately after device deployment with almost complete resolution of shunt by the next day. However, the multi-institutional US Registry study ^80^ reports a complete closure rate of 47% at 24 hours after the procedure, which increased to 70% at 6 months and 92% at 12 months after the procedure. Reported complications include transient complete left bundle branch block; transient junctional rhythm; complete heart block, both transient and permanent (the latter requiring pacemaker implantation); tamponade resulting in death; cardiac perforation; device embolization (some retrieved by transcatheter methodology and others by surgery), device malposition requiring surgical removal; severe hemolysis (some causing renal failure); and death ^34, 36, 38, 74, 80– 84^; however, these complications are rare. Follow-up results were reported in a few studies but with no additional complications.

***Hybrid (perventricular) device delivery.*** Because of inherent limitations of both surgical and percutaneous device closure of muscular VSDs in small infants, a hybrid approach, also called “perventricular closure”, was introduced ^85, 86^. The initial descriptions included six infants ^85^ and seven mini pigs ^86^. Feasibility of this approach was demonstrated in both young infants and animal models without cardiopulmonary bypass ^85, 86^. A number of other investigators ^87– 101^ subsequently adopted this technique.

**The procedure**

Under general anesthesia, a median sternotomy or a subxiphoid incision is made and the right ventricular free wall is needle-punctured via a purse-string suture. The soft end of a guide wire is introduced across the VSD into the LV under TEE guidance. The needle is removed and an appropriate-size delivery sheath with a dilator is advanced over the guide wire into the LV and the dilator and guide wire are withdrawn, leaving the tip of the sheath in the LV. An appropriate-size device (1 to 2 mm larger than the TEE diameter of the VSD) is advanced through the sheath. Once the device reaches the tip of the sheath, the sheath is retracted to extrude the left ventricular disc in the mid LV. Then the entire system is slowly withdrawn so that the LV disc is apposed to the interventricular septum. Further slow retraction of the sheath is undertaken, delivering the device waist within the defect and the right ventricular disc on the right side of the ventricular septum—all while the procedure is being monitored by TEE and while the heart is beating. Once the position of the device is satisfactory as evaluated by TEE, the delivery wire is disconnected and the sheath is removed while the purse-string suture is tightened. A final TEE is performed to ensure appropriate position of the device and to detect any additional VSDs.

**Results**

Immediate results of hybrid procedures to close perimembranosus and muscular VSDs have been reported ^85– 101^. The number of subjects included was anywhere between 6 and 408 patients. Successful device implantation is reported from 82 to 100% of patients. In most studies, unsuccessful implantations were converted to conventional open-heart surgical repair, irrespective of the reason for failure. Residual shunts were not significant. Complications were few and the occasional pericardial effusion occurred.

In one large study involving 408 patients with perimembranous VSDs with a mean age of 3.1 years (± 1.7 years), an age range of 5 months to 15 years, and a weight range of 4.5 to 26 kg, successful closure was accomplished in 393 patients (96.3%) ^90^. The devices used were similar to Amplatzer VSD devices but manufactured in China by the Shanghai Alloy Material Corporation; 213 (54.2%) were symmetric devices and 180 (45.8%) were asymmetric devices. Follow-up from three months to two years after the procedure exhibited no residual shunts, stable device position, and no increase in aortic insufficiency.

In a multicenter retrospective study from Europe, investigators reported closure of muscular VSDs in 21 patients; the device was successfully implanted in 89% of patients ^91^. In the remaining patients, the device was removed because of arrhythmia, malposition, or additional defects. At a mean follow-up of 1.4 years, only one patient had more than trivial shunt. No other complications occurred.

Since the perimembranous VSD closure with devices is likely to result in heart block as described in the “Percutaneous closure” section, we do not advocate their closure by a hybrid procedure. Detailed review of these hybrid procedure reports reveals that many patients had small VSDs (<5 mm) or aneurysmal formation of membranous ventricular septum, a natural attempt for spontaneous closure, and the authors of this article question the advisability of closure of such VSDs.

This hybrid technique is especially helpful in small babies with large muscular VSDs in whom there is a higher prevalence of adverse events for percutaneous device closure when compared with older children.

### Comparison of various methods of intervention to occlude ventricular septal defects

There are a limited number of studies comparing one method of VSD closure with the other.

***Percutaneous versus surgical closure.*** In one study, the results of percutaneous closure in 852 patients were compared with 1,326 patients who had conventional surgical repair ^102^. Procedure success rates and prevalence of major complications were similar. However, the rates of minor complications (6.4% versus 0.6%) and requirement of blood transfusions (10.3% versus 0%) and duration of hospitalization (12.9 versus 3.2 days) were higher in the surgical than in the percutaneous group. The authors concluded that the percutaneous approach is an effective and reasonable alternative to surgery in the management of patients with VSD ^102^.

In another study, the results of percutaneous versus surgical closure of perimembranous VSDs were compared by examining the results of seven previously published articles; there were 1,312 percutaneous device implantations and 1,822 surgical closures ^103^. The patients in the percutaneous group were older than those in the surgical group (mean age of 12.2 versus 5.5 years), but the VSD sizes were similar. Procedural success rates, major complications such as need for reoperation, early deaths, and requirement for permanent pacemaker implantation were similar as were the residual shunts, significant aortic and tricuspid insufficiency, and advanced heart block at follow-up. The higher requirement for blood transfusion and longer duration of hospital stay were seen in the surgical group. The authors concluded that percutaneous intervention and surgical closure of perimembranous VSDs had similar procedural success rates and that percutaneous closure did not manifest higher rates of valvar insufficiency and heart block than surgical closure ^103^.

***Hybrid versus surgical closure.*** A comparison of 49 hybrid device closures with 41 surgical closures was made ^98^. The ages were similar, but the VSDs were slightly larger (6.03 versus 5.03 mm) in the surgical group. Major complications, namely death, severe valvar insufficiency, significant residual shunts, and lethal arrhythmias, did not occur in either group. Complete closure rates immediately after procedure and at follow-up were similar for the two groups. The requirement for blood transfusion was higher and the length of the stay in intensive care unit was longer in the surgical than in the hybrid group. The authors conclude that hybrid VSD closure may be an alternative to surgical closure ^98^.

***Comments on comparisons.*** Although the authors of the above studies have made considerable efforts to compare various groups, all studies appear to be retrospective and non-randomized and may not be as definitive as the authors would like us to believe. In addition, it should be observed that the above-mentioned comparative studies do not compare identical patient populations and there is a selection bias in the patients managed with device closure because only ideal candidates are managed with device closures, and more complex VSDs were referred for surgery. Additionally, they do not take into account that repair under cardiopulmonary bypass will entail a period of ventilation and intensive care stay.

### Conclusions

When one carefully examines the data of patients undergoing VSD closure, many VSDs are less than 5 mm in size and the Qp:Qs was less than 2:1. The availability of less invasive transcatheter approaches should not, in the authors’ opinion, relax the indications for closure and these indications should be the same as those used for standard surgical closure. In addition, the natural history studies indicate that spontaneous closure occurs in VSDs and such closures continue to occur during childhood, adolescence, and adulthood. The pediatricians and the general pediatric cardiologists should serve as gatekeepers to prevent intervention by pediatric cardiac surgeons and interventional pediatric cardiologists for “small” VSDs that do not strictly fit established criteria for closure, irrespective of the type of intervention, whether it is conventional surgical, percutaneous, or hybrid.

Based on clinical experience for over 45 years in caring for patients with VSDs by the senior author (PSR) and an extensive review of the subject at the time of this writing, the following recommendations may be made. Open-heart surgical closure remains the main treatment option for large and non-restrictive perimembranous VSDs. Timely intervention to prevent PVOD is highly important and is in the purview of the pediatrician and pediatric cardiologist caring for the child. Percutaneous closure seems to be a valuable option for closure of large muscular VSDs. A hybrid procedure is a good option for large muscular VSDs in small infants. When that is not possible, particularly in babies with the Swiss-cheese type of VSDs, banding of the pulmonary artery as an initial palliative procedure with later closure of VSD is likely to be beneficial to these infants.

## Atrioventricular septal defects

What were formerly known as AV canals and endocardial cushion defects are now termed as atrioventricular septal defects (AVSDs). The characteristic features of this CHD include defects in atrial and ventricular septa along with deficiency in one or both AV valves. The AVSDs constitute 4 to 5% of all CHDs. The AVSD is the most common defect in babies with Down syndrome.

The AVSDs are classified into partial, transitional, intermediate, and complete forms ^104^. The partial form usually had a large ASD in the anterior portion of the lower part of the atrial septum along with a cleft in the anterior leaflet of the mitral valve. A cleft in the septal leaflet of the tricuspid valve may also be present in some patients. In the transitional form, there is an additional small inlet VSD and the physiology is similar to that of the partial form. The management of the partial form, also called ostium primum ASD, and transitional form is similar and was discussed in our previous review ^1^ and will not be repeated here. The complete form has one AV valve annulus, a large ostium primum ASD, and a contiguous large inlet VSD. The intermediate form is similar to the complete form with the exception that the single AV annulus is divided into two orifices by a tongue of tissue. The complete forms are further divided into Rastelli types A, B, and C on the basis of the characteristics of the anterior bridging leaflet ^105^. Another classification based on relative ventricular sizes is balanced and unbalanced AVSDs; the unbalanced defects constitute 10 to 15% of all complete forms of AVSDs. The unbalanced forms may be LV-dominant (large LV and small RV) or RV-dominant (large RV and small LV); the RV-dominant AVSDs are more common. The unbalanced AVSDs require different types of surgical approaches.

Hemodynamic abnormalities in patients with AVSD are secondary to the left-to-right shunt across the ASD and VSD components and mitral valve insufficiency. As mentioned in the “Ventricular septal defects” section, the left-to-right shunt across the ASD and VSD may not manifest in the neonate and young infant and this is due to high PVR. As the pulmonary arterioles involute and PVR falls, left-to-right shunt occurs, resulting in dilatation of the left atrium and LV; this may take several weeks unless there is an anomalous and rapid fall in PVR. Because the ASD and VSD components are large, there is dilatation of the right atrium, RV, and main and branch pulmonary arteries. If there is moderate to severe mitral insufficiency, further dilatation of the left atrium and LV ensues. PVOD may develop as early as six months to one year of age in patients with complete AVSDs and even earlier in babies with Down syndrome.

Discussion of AVSDs seen in association with tetralogy of Fallot, pulmonary atresia/stenosis, transposition of the great arteries, and double-outlet RV will not be included in this review.

### Indications for atrioventricular septal defect repair

Most, if not all, complete and intermediate types of AVSD have large ASD and VSD components and therefore all patients with AVSD should have their defects repaired. Alternatively, pulmonary artery banding ^106^ may be performed. At present, primary complete repair is performed at most institutions. Pulmonary artery banding may be considered for babies weighing less than 5 kg in whom the CHF could not be controlled and babies with other significant co-morbidities ^107– 109^.

In patients with elevated PVR, the considerations for operability ^6– 8^ and pulmonary vascular reactivity testing ^6, 8, 9^ are similar to those described in the “Ventricular septal defects” section and will not be repeated. Patients with AVSDs and severely elevated PVR (that is, irreversible PVOD) are not suitable for AVSD repair. These patients eventually may become candidates for lung transplantation.

### Management

Medical and surgical treatment options for balanced and unbalanced AVSDs will be reviewed separately.

***Balanced atrioventricular septal defects (complete and intermediate).***

**Medical management**

Neonates with AVSDs with no signs of CHF do not need treatment in the newborn period; however, we usually begin treatment with infrequent doses of furosemide (once every other day) since a predictable decrease of PVR with time occurs with a resultant increase in pulmonary blood flow and development of CHF. In a few weeks, these infants will develop CHF; at that time, they should be managed with aggressive anticongestive treatment, as reviewed in the “Ventricular septal defects” section above, including optimization of nutrition, maintenance of adequate hemoglobin level, and appropriately addressing the associated respiratory symptoms.

Similar to what was discussed in the preceding section on VSDs, patients with a severe increase of PVR (irreversible PVOD) should not have their AVSDs closed. The management of PVOD associated with AVSDs is the same as that discussed in the VSD section.

**Surgical management**

In order to prevent the development of irreversible PVOD, timely surgical repair, preferably prior to six months of age, should be performed. Although it is our general impression that babies with Down syndrome develop PVOD earlier than non-Down babies, histological studies do not seem to indicate any difference in pulmonary vascular changes between Down and non-Down children ^110^. Pulmonary alveolar and capillary hypoplasia ^111^ and chronic upper airway obstruction with resultant hypoventilation producing hypoxia and hypercarbia may be responsible in part for higher PVR ^112^ and early PVOD in babies with Down syndrome.

**Corrective surgery.** Surgical correction is performed after placing the patient on cardiopulmonary bypass and consists of patch closure of atrial and ventricular septal defects along with repair and reconstruction of AV valves. Closure of ASD and VSD components may be performed by using either a single-patch technique (pericardial) or two-patch technique (pericardial patch for primum ASD closure and pericardial or Dacron patch for the VSD). In all instances, the common AV valve is separated into left and right components; the left AV valve cleft is completely or partially closed on the basis of subvalvar anatomy, and the right AV valve cleft may be closed as indicated. In some instances, the Australian repair (primary closure of VSD component with septation of the common AV valve, closure of AV valve cleft, primum ASD closure with pericardial patch) may be considered. A recent metal analysis comparing single-patch with two-patch technique demonstrated no significant difference between two groups with comparable outcomes ^113^; however, the cardiopulmonary bypass and aortic cross-clamp times were shorter in the single-patch method of closure of the defect ^113^. AV valves are repaired/reconstructed by using the techniques described by Carpentier ^114^, Puga and McGoon ^115^, or Chopra *et al*. ^116^. Associated defects such as patent ductus arteriosus and left ventricular outflow tract obstruction (usually fibromuscular membrane) should be addressed at the same sitting. In the present day, TEE is performed to evaluate mitral and tricuspid valve function (mitral/tricuspid insufficiency and stenosis) and residual shunts prior to decannuation from cardiopulmonary bypass. Re-repair of mitral (or, in rare cases, tricuspid) valve is generally undertaken if the degree of insufficiency or stenosis is more than mild.

**Pulmonary artery banding.** As mentioned above, banding of the pulmonary artery may be performed in babies weighing less than 5 kg in whom the CHF could not be controlled and when associated with significant co-morbidities. The banding should be tight enough to produce near normal pressures distal to the band, but care should be taken to avoid any deterioration in LV function based on intra-operative transesophageal echocardiogram. Corrective surgery along with the removal of the band may be performed a few months later when the infant improves clinically. However, the trend is for complete repair at most institutions.

**Surgical results**

A large number of investigators reported results of surgical repair of AVSDs. We examined the results of some of these studies ^116– 125^, which included 37 to 1,917 patients. The immediate mortality rates varied between 2 and 62% ^116– 125^. Some studies compared the results of the earlier era with those of the present time, these comparisons have consistently shown improvement of mortality rates in recent years. More recent studies demonstrated a substantial fall in mortality rates (down to 2 to 3%) ^122, 124, 125^. Although there are differences in risk factors for surgical mortality from one study to the next, the most commonly found risk factors were preoperative severity of mitral regurgitation and New York Heart Association (NYHA) functional class. Other risk factors identified in some studies were very young age, small size of the babies, postoperative residual mitral valve regurgitation, ventricular hypoplasia (unbalanced ventricular sizes), additional muscular VSDs, and double-orifice mitral valve.

There is some early concern regarding poor tolerance for surgery in the patients with Down syndrome. One relatively recent study ^126^ compared the outcomes and complications between Down and non-Down babies. There was no postoperative mortality in either group. Cardiac complications such as the prevalence of junctional ectopic tachycardia, the need for early reoperation, and the requirement for insertion of a permanent pacemaker for complete heart block were similar in the two groups. However, there was higher prevalence of non-cardiac complications such as pneumothorax, pleural effusions, and infections in infants with Down syndrome than in those without Down syndrome. However, these non-cardiac complications did not increase duration of stay in the pediatric intensive care unit. These data affirm our thinking that babies with Down syndrome should receive the same treatment as those without. In fact, non-Down babies tend to have more abnormal left AV valve morphology, and AVSD repair in children with Down syndrome is more straightforward than that in babies without Down syndrome. In the current era, surgery is the therapy of choice in all infants and ideally is performed between three and six months of age.

In studies in which long-term follow-up data were available, actuarial survival rates were reported be 91%, 91%, and 89% at 1, 5, and 15 years, respectively ^123^, and 85%, 82%, and 71% at 10, 20, and 30 years, respectively ^124^. The estimated rates of freedom from late reoperation were 96% at 1 year, 89% at 5 years, and 89% at 15 years ^123^. Reoperation may be required in 10 to 15% of patients; mitral valve regurgitation (7 to 10%) is the most common reason for late reoperation, followed by left ventricular outflow tract obstruction (3.5%). A small percentage of patients may require surgery for tricuspid regurgitation, mitral stenosis, or residual shunts. Risk factors for late reoperation were other cardiovascular anomalies, mitral valve dysplasia, and absence of mitral cleft closure during the initial surgery ^123^.

***Unbalanced atrioventricular septal defects.***

**Medical management**

The medical management at presentation is similar to that described for balanced AVSDs.

**Surgical management**

Several surgical options are in use for treating unbalanced AVSDs and will be reviewed. 

**Single-ventricle palliation (Fontan)**

The reparative techniques described above for balanced AVSDs cannot be used in unbalanced AVSDs because of hypoplasia of the RVs or LVs which after the repair may not be able to support the pulmonary or systemic circulations, respectively. Marginal-sized RVs may be addressed with a combination of biventricular repair and bidirectional Glenn procedure. By and large, these patients require a staged Fontan surgery similar to that described for tricuspid atresia and other single-ventricle (SV) lesions ^125– 131^. Remarkable changes in the concepts and methods of surgery have occurred since the initial description of right ventricular bypass by a complex procedure by Fontan ^125^ and a simpler atriopulmonary anastamosis by Kruetzer ^126^. The descriptions of bidirectional Glenn ^132– 134^, total cavopulmonary connection ^135^, extracardiac non-valved conduits ^136, 137^, staged procedures ^133, 134^, creation of fenestration ^138– 140^, and device closure of fenestration ^139^ have resulted in the evolution of the procedure such that it is now a staged (three-stage) total cavopulmonary connection with an extracardiac conduit and fenestration with subsequent device closure of the fenestration ^129– 131^.

Following control of CHF, as described above in the “Balanced atrioventricular septal defects” section, pulmonary artery banding is performed to limit the pulmonary blood flow, control CHF, and normalize the pulmonary artery pressures (stage I of Fontan). At about six months of age, a bidirectional Glenn procedure is performed (stage II of Fontan). Prior to the procedure, one must be assured of normal pulmonary artery pressures; if this cannot be done by echo-Doppler studies, cardiac catheterization may be required. In the presence of a persistent left superior vena cava, a bilateral, bidirectional Glenn procedure may be required, especially if the bridging left innominate vein is absent or small. If there is significant AV valve regurgitation or other hemodynamically significant abnormalities, they should be appropriately addressed at this stage. One year after the bidirectional Glenn (or between one and four years of age), the inferior vena caval flow is diverted into the pulmonary artery by either a lateral tunnel or an extracardiac non-valved conduit (stage IIIA); most surgeons seem to prefer extracardiac conduit with fenestration. Cardiac catheterization and selective cine-angiography are recommended (to evaluate pulmonary artery anatomy and pressures, transpulmonary gradient, PVR, and left ventricular end-diastolic pressure) prior to stages II and III to ensure suitability for proceeding with the next stage of surgery. Six months to one year after stage IIIA, the fenestration is closed percutaneously with a device (stage IIIB). Detailed angiographic anatomy at each Fontan stage is demonstrated in a prior publication ^131^, and the interested reader is referred to that article.

**Results of single-ventricle palliation (Fontan).** The operative mortality in patients who had staged, cavopulmonary connection with fenestration appears to be between 4.5 and 7.5% ^131, 141^. On follow-up, actuarial survival rates were 93% at 5 years and 91% at 10 years ^142^. In another study, the actuarial survival was 85% at 15 years ^143^. The re-intervention (surgical or percutaneous) rate was 12.7%. It should be noted that the patients in these studies included all types of SV lesions, namely tricuspid atresia, double-inlet left (single) ventricle, hypoplastic left heart syndrome, mitral atresia with normal aortic root, unbalanced complete AVSDs, pulmonary atresia with intact ventricular septum with markedly hypoplastic RV, and any complex heart defect with one functioning ventricle. When one examines Fontan procedures in patients with unbalanced AVSDs, the mortality rates (17 to 31.7%) were higher ^144, 145^ and actuarial survival rates (66.5% at 5 years and 64.4% at 15 years) were lower ^146^ and there were more surgical and catheter re-interventions (51.9%) ^145^. Equally poor results with an overall long-term survival rate of 50% were observed in another study; these results were poorer than for a cohort with hypoplastic left heart syndrome patients operated during the same period ^146^. It should be noted that complications, namely arrhythmias, Fontan pathway obstruction, cyanosis, paradoxical emboli, formation of thrombus, collateral vessels, and protein-losing enteropathy, may be seen on long-term follow-up, although most of these complication are less common with staged total cavo-pulmonary connection than with earlier versions of Fontan ^130, 131^.

**Two-ventricle repair**

The AV valves are repaired to create nearly equal-sized AV valves along with complete (single stage) or partial (staged) closure of ASD and VSD components ^144, 147– 151^. In the staged procedure, the residual defects are repaired surgically after demonstration of growth of the hypoplastic ventricle.

The criteria used to select a given patient to SV versus two-ventricle (TV) route are not clearly established. Several investigators examined this issue by evaluating potential echocardiographic LV volume after normalization of septal bowing, ventricular cavity ratio between the two ventricles (estimated as left ventricular length × width/right ventricular length × width), AV valve index (left AV valve area/total AV valve area), a different type of AV valve index (left valve area/right valve area), ventricular dimensions, LV end-diastolic volume index, RV/LV inflow angle in systole, left AV valve color diameter at smallest inflow, left AV valve color diameter at annulus, indexed ventricular septal defect (defined as the size of the defect in relation to the common AV valve diameter), left ventricular inflow index (calculated as the secondary color inflow diameter indexed to the left AV valve annulus diameter), and other indices ^145, 151– 159^.

At present, no clear-cut, uniform echocardiographic criteria are established for selecting a given unbalanced AVSD patient for TV repair. However, some investigators advocate TV repair if AV valve index (left/right valve area) is more than 0.5 ^149^, more than 0.65 ^150^, or more than 0.67 ^152^ for right dominant unbalanced AVSDs. Other suggestions for selecting TV repair are indexed potential LV volume of more than 15 mL/m ^2^ ^151^, left ventricular inflow index greater than 0.5 ^154^, and indexed ventricular septal defect smaller than 0.2 ^158^. The last group of investigators ^158^ also recommended that patients with indexed VSD values between 0.2 and 0.35 may be considered for TV repair on the basis of other factors. If the indexed VSD value is between 0.35 and 0.5, SV palliation may be required. Other investigators examined various echo-Doppler parameters and made a correlation between these parameters and survivals but did not include definitive cutoff points for TV repair. Another group of investigators used angiographic measures and suggested that patients with LV-to-RV long axis ratio of more than 0.65 on cine-angiograms may be suitable for TV repair ^150^. Perhaps a combination of indexed VSD, AV valve index, and RV/LV inflow angle in systole may guide the selection of patients for TV repair ^158, 160^.

**Results of two-ventricle repair.** There are limited data with regard to the results of TV repair. The mortality rates varied between 10.4 and 18% ^144, 147, 153^, although it was a higher (40%) in an earlier series ^152^. Long-term survival was good and ranged from 88 to 90% ^144, 147, 150^. However, surgical (17.4 to 34%) or transcatheter (13.4%) re-interventions were frequently required ^144, 147, 150^. But improvement of AV valve Z-scores (−2.8 to −7.4 versus −0.6 to −2.7 ) ^147^, ventricular dimension Z-scores (−1.0 to −7.5 versus −2.0 to +1.8) ^147^, and LV end-diastolic volume Z-scores (from a median of −3.15 to +0.42) ^144^ occurred at follow-up.

**Conversion from single-ventricle to two-ventricle repair**

This group involves patients who initially had SV palliation but were later converted to TV circulation. This approach was precipitated by suboptimal long-term outcomes after SV palliation ^159^. Rehabilitation of the left heart is undertaken by promotion of flow through the LV by relief of inflow or outflow tract obstructions (or both) and resection of endocardial fibroelastosis (if present). Restriction of the atrial septum to promote flow through the LV is also undertaken. 

**Results of conversion from single-ventricle to two-ventricle repair.** The reported mortality following conversion to TV circulation is low (1 to 11%) ^144, 161^. Surgical (19%) and catheter (38%) re-interventions were required during follow-up ^161^. Although the initial Z-scores of the LV prior to stage I SV palliation were not significantly different, the LV Z-scores increased significantly in the SV to TV group but decreased in the SV palliation group without conversion ^159^. Another study from the same institution ^162^ demonstrated improvement of left ventricular end-diastolic volume by echocardiography from 28.1 to 58.5 mL/m ^2^ in a small group of the unbalanced AVSD group following SV-to-TV conversion ^162^.

A recent study examined mid-term outcomes of primary and staged biventricular repair and SV-to-TV conversion of unbalanced complete AVSDs and concluded that these methods result in low morbidity and mortality although re-interventions (both surgical and catheter) in 52% of patients were required ^145^.

**Comparison of various methods of intervention for unbalanced atrioventricular septal defects**

Nathan *et al*. compared the outcomes of patient cohorts who had SV palliation, TV repair, and conversion from single-ventricle to two-ventricle (SV to TV) ^144^. The TV repair and SV-to-TV groups were reasonably similar with regard to mortality and the need for heart transplantation but were lower than that seen with the SV palliation cohort during a mean follow-up of 35 months. Although the surgical re-interventions were similar in the three groups, catheter re-interventions were lower in the TV repair group than in the other two groups. They conclude that TV repair and SV-to-TV conversion may be accomplished with lower mortality and morbidity rates in children with unbalanced AVSDs, although the study was critiqued ^163^ for allowing survivorship bias in favor of TV repair and SV-to-TV groups. Conversion from SV palliation and biventricular recruitment remains an important option for children with Down syndrome and heterotaxy syndrome for physiologic and anatomic reasons. Patients with Down syndrome tolerate SV physiology poorly ^164^. Patients with heterotaxy syndrome have complex and abnormal AV valve anatomy and require complex and innovative repair techniques. They also tolerate AV valve regurgitation poorly when palliated via the SV pathway.

## Conclusions

Treatment of CHF (if present) followed by surgical repair with closure of atrial and ventricular septal defects along with repair and reconstruction of AV valves is a standard approach in babies with balanced AVSDs. Pulmonary artery banding may be performed to palliate infants weighing less than 5 kg as well as those with significant co-morbidities. Timely intervention to prevent PVOD is exceedingly important. The management of unbalanced AVSDs is complex; staged SV palliation (Fontan) is performed at most institutions, but recent data seem to indicate that primary or staged TV repair or converting SV to TV repair may be better choices. The management of babies with Down syndrome should be similar to that used for non-Down infants.
